# Design of Organoiron Dendrimers Containing Paracetamol for Enhanced Antibacterial Efficacy

**DOI:** 10.3390/molecules25194514

**Published:** 2020-10-02

**Authors:** Alaa S. Abd-El-Aziz, Ebtehal G. El-Ghezlani, Amani A. Abdelghani

**Affiliations:** Department of Chemistry, University of Prince Edward Island, 550 University Avenue, Charlottetown, PE C1A 4P3, Canada; eel@upei.ca (E.G.E.-G.); aabdelghani@upei.ca (A.A.A.)

**Keywords:** dendrimer, paracetamol, organoiron, redox-active, MRSA, VRE, *staphylococcus warneri*, SEM

## Abstract

Paracetamol (acetaminophen) is a common painkiller and antipyretic drug used globally. Attachment of paracetamol to a series of organoiron dendrimers was successfully synthesized. The aim of this study is to combine the benefits of the presence of these redox-active organoiron dendrimers, their antimicrobial activities against some human pathogenic Gram-positive, and the therapeutic characteristics of paracetamol. The antimicrobial activity of these dendrimers was investigated and tested with a minimum inhibitory concentration and this has been reported. Some of these newly synthesized dendrimers exhibited the highest inhibitory activity against methicillin-resistant *Staphylococcus aureus* (MRSA), vancomycin-resistant *Enterococcus faecium* (VRE), and *Staphylococcus warneri* compared to reference drugs. The results of this study indicate that the antimicrobial efficacy of the dendrimers is dependent on the size of the redox-active organoiron dendrimer and its terminal functionalities. The best result has been recorded for the fourth-generation dendrimer **11**, which attached to 48 paracetamol end groups and has 90 units composed of the η^6^-aryl-η^5^-cyclopentadienyliron (II) complex. This dendrimer presented inhibition of 50% of the growth (IC_50_) of 0.52 μM for MRSA, 1.02 μM for VRE, and 0.73 μM for *Staphylococcus warneri.* The structures of the dendrimers were characterized by elemental analysis, Fourier transform infrared (FT-IR), nuclear magnetic resonance (^1^H-NMR), and ^13^C-NMR spectroscopic techniques. In addition, all synthesized dendrimers displayed good thermal stability in the range of 300–350 °C following the degradation of the cationic iron moieties which occurred around 200 °C.

## 1. Introduction

Multidrug-resistant diseases are a global burden with a continuous loss of human life predicted for the future [1]. While careful use of antimicrobials provides a possible solution, the development of novel types of antimicrobials with different mechanisms of action is important to help in the fight against the rise of multidrug-resistant infections such as methicillin-resistant *Staphyloccus aureus* (MRSA) and vancomycin-resistant *Enterococcus faecium* (VRE). As a result, synthesis and investigation of antimicrobial molecules and macromolecules has been given a great deal of interest [2,3,4,5,6]. Indeed, these macromolecules have been used for the treatment and inhibition of multidrug-resistant infections [2,3,4,5,6]. Dendrimers are the best class of these macromolecules as their structures can be intelligently designed to improve the activity and increase selectivity [6]. Dendrimers are unique highly branched 3-dimensional macromolecules that arise from a central core [7,8,9,10]. Several unique properties associated with dendrimers allows them to be used in many applications such as catalysis [11,12,13,14], sensing [15,16,17], and fluorescence applications [18,19,20,21,22], General synthesis routes to these dendrimers involve functionalizing the periphery with antimicrobial agents [6]. The search for unique synthesis approaches of antimicrobial dendrimers is expanding with the focus on enhanced activity upon resistant strains [15,16,23,24,25,26,27,28,29,30,31,32,33,34,35,36]. The advantage of including η^6^-aryl-η^5^-cyclopentadienyliron (II) complexes into dendritic structures is to provide highly-ordered structures with engaging bioactivity and other properties [16,37,38]. Doubtless, the association of organo-transition metal groups within dendritic structures has attracted attention in both organometallic and dendrimer research and opened up a new family of organometallic macromolecules with many novel properties [39,40,41,42]. The complex, as well as its dendrimer derivatives, were confirmed in our previous studies to display antimicrobial activity [33,43].

The first published use of paracetamol (N-acetyl-*p*-aminophenol, also commonly known as acetaminophen) for therapeutic purposes in humans was by Von Mering in 1893, but it was not generally used until the 1950s [44]. Now, paracetamol is the most important coal tar derivative analgesic drug, due to its minor toxicity which allows it to be used more widely. Despite the popularity of this drug, the mechanism by which paracetamol achieves its effects on fever and pain is still under discussion [45]. It could be through the activation of descending serotonergic pathways, due to the inhibition of prostaglandin (PG) synthesis, or through an active metabolite affecting cannabinoid receptors [45]. Due to the similarity between paracetamol and aspirin in analgesic and antipyretic non-steroidal anti-inflammatory drugs (NSAIDs), a great deal of research has centered on paracetamol inhibition of the Cyclooxygenase (COX) enzyme, however, paracetamol does not have significant anti-inflammatory activity [46].

While paracetamol does allow for a reduction of morphine use, thereby diminishing morphine-related opposing effects [47,48,49,50], the implicit principle is that the distinct modes of action of morphine and paracetamol provide maximum analgesia to be managed with a lower dose of morphine, and, consequently, a smaller percentage of morphine-related adverse effects occur [47,48,49,50].

In this work, we tested the antimicrobial activity of a series of cationic, redox-active dendrimers against microorganisms that included MRSA, VRE, and *Staphylococcus warneri*. The primary properties of macromolecules such as thermal stability using thermogravimetric analysis (TGA) and electrochemistry using cyclic voltammetry (CV) were also investigated. The results show that the antimicrobial dendrimers generated free radicals, had good thermal stability, and exerted an excellent antimicrobial activity against multidrug-resistant bacteria. In addition, the dendrimeric structure was approved by spectroscopic and elemental analyses, which were also utilized to differentiate between the four generations of dendrimers as well as terminal end groups of the same generation. The determination of the morphology of dried residues of the dendrimers was also performed using scanning electron microscopy (SEM).

## 2. Results and Discussion

The purpose of this article was the design of an organometallic dendrimer with a painkiller and tunable antimicrobial activity. Four dendrimers were functionalized with paracetamol in the first, second, third, and fourth generations. To achieve this, the redox-active organoiron moieties were incorporated throughout every dendritic branch.

### 2.1. Syntheses and Characterization of the Complexes

**Scheme 1:**
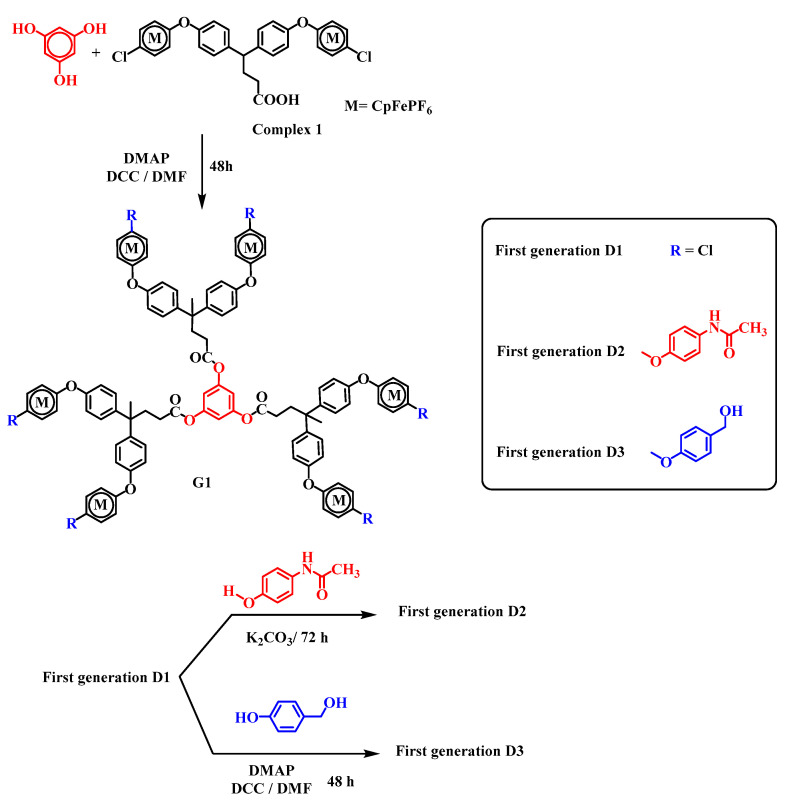
Schematic representation of the synthesis of first-generation dendrimers (**D1–D3**).

**Scheme 2:**
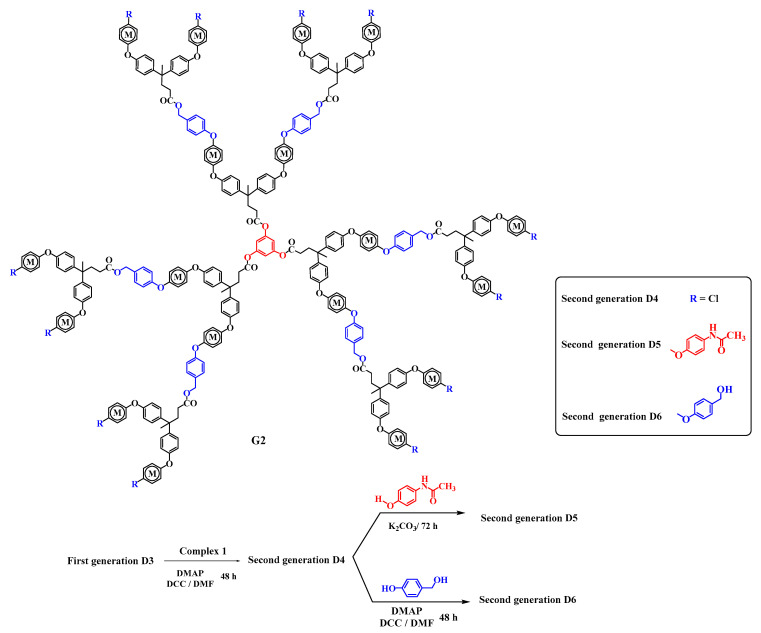
Schematic representation of the synthesis of second-generation dendrimers (**D4–D6**).

**Scheme 3:**
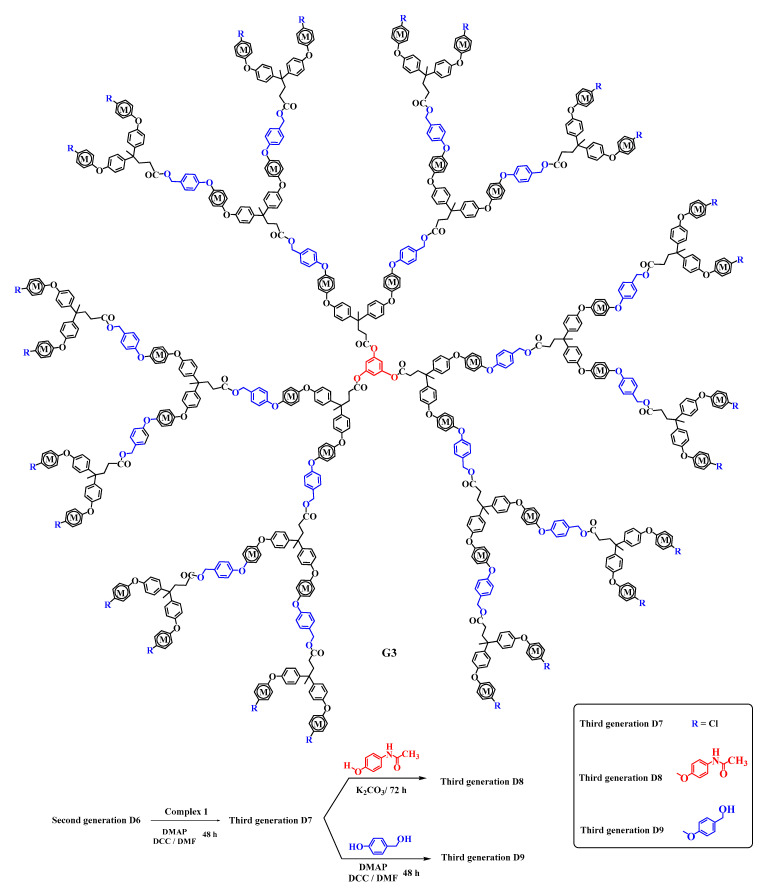
Schematic representation of the synthesis of third-generation dendrimers (**D7–D9**).

**Scheme 4:**
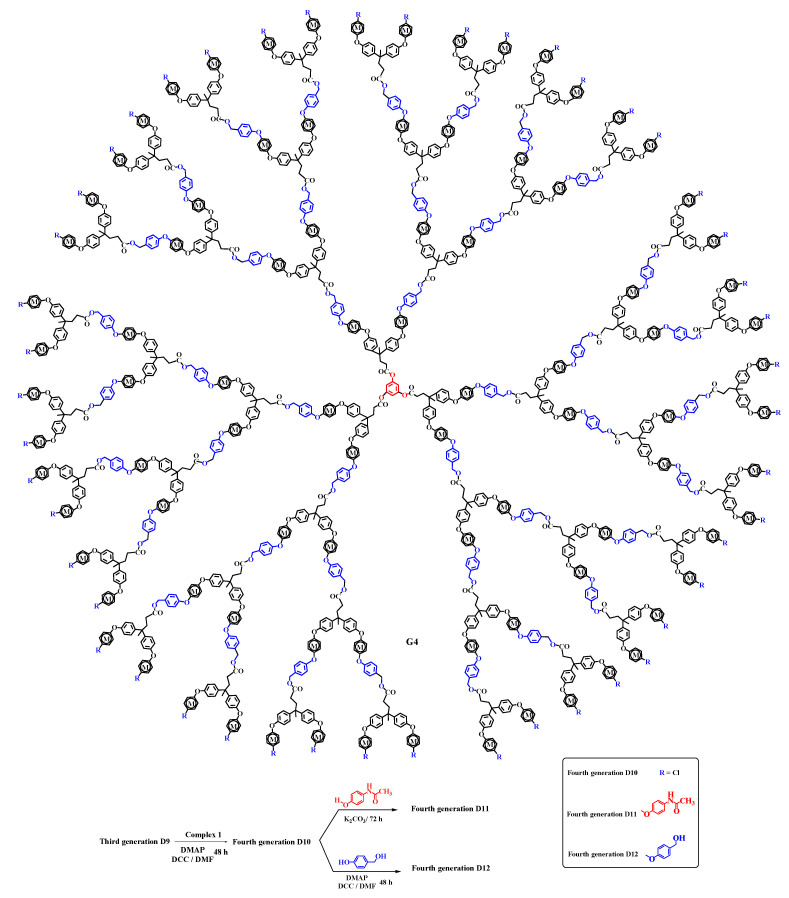
Schematic representation of the synthesis of fourth-generation dendrimers (**D10–D12**).

All dendrimers were synthesized from cationic η^6^-arene-η^5^-cyclopentadienyliron(II) (Cp-Fe^II^-arene) complexes of PF_6_^–^ counteranions using a common synthetic route previously described here and presented in Scheme 1, Scheme 2, Scheme 3 and Scheme 4. The chemistry of the complex allows easy functionalization of the periphery of the dendrimers with the painkiller paracetamol, producing antimicrobial organometallic dendrimers with central analgesia properties. For example, the synthesis of the **first-generation D1**, **second-generation D4**, **third-generation D7**, and **fourth-generation D10**, involved esterification of the carboxylic groups of complex **1** with the hydroxy group, to form the first set of dendrimers with chloro end group. The nucleophilic substitution reaction of these dendrimers with paracetamol **2**, leads to the second set of dendrimers, **first-generation D2**, **second-generation D5**, **third-generation D8**, and **fourth-generation D11**, with paracetamol end group. The last set of dendrimers were used 4-hydroxybenzyl alcohol **3** to form **first-generation D3**, **second-generation D6**, **third-generation D9**, and **fourth-generation D12**, with the hydroxyl end group, by the nucleophilic substitution reaction as well.

^1^H and ^13^C NMR and infrared (IR) spectroscopy as well as elemental analysis were used to characterize and identify the new dendrimers. For instance, **second-generation D4** showed a downfield peak at 6.82 ppm, which referred to 24 protons in the twelve complexed outer aryl groups attached to the chloro end groups, and another upfield peak at 6.43 ppm, which represented 24 protons in the 12 complexed outer aryl groups attached to the etheric oxygen groups plus the 24 protons of the six iron-complexes in the first layer of the dendrimer. Additionally, a peak appeared at 5.28 ppm, which corresponded to the protons in the Cp attached to the chloro- end groups and another peak at 5.22 ppm, which corresponding to the 6 inner cyclopentadiene complexes. The **second-generation** **D5** showed one upfield peak appearing at 6.25 ppm; the upfield peak was indicative of the protons close to the peripheral attached to the etheric oxygen groups. The equivalent protons of the iron-complexed arene ligand, indicated a successful S_N_Ar reaction since, as discussed in the literature [20,34,51], a successful S_N_Ar reaction with phenolic nucleophiles converts the non-equivalent aromatic protons of iron-complexed chlorophenoxyl into equivalent protons. The (NH) peak of secondary amine groups in paracetamol appeared at 10.13 ppm. The Cp peak shifted to the upfield region and appeared at 5.22 ppm, corresponded to the protons close to the etheric oxygen groups which replaced the chloro end groups. Also, in ^1^H NMR spectroscopy the appearance of two Cp peaks again at 5.28 and 5.22 ppm for **third-generation** **D7**, was due to the non-equivalence of the surrounding groups at the para positions. The first peak at 5.22 ppm corresponded to the inner protons close to the etheric oxygen groups while the second peak shifted downfield at 5.28 ppm, due to the presence of the chloro end group. It is also worth noting that both Cp peaks showed integration in agreement with the ratio of Cp protons pendent to the chloro-arenes in the periphery to those in the inner arenes with the etheric bridges. Also, of the three denoted peaks at 6.81, 6.40, and 6.26 ppm, the upfield peak at 6.26 ppm referred to the 72 protons in the eighteen inner complexed aryl groups attached to the etheric oxygen groups, while the other peaks observed at 6.82 and 6.43 ppm corresponded to the 96 protons in the 24 complexed outer aryl rings with the chloro end groups. In addition, the OH peak at 5.33 ppm in **second-generation** **D6** disappeared in the **third-generation** **D7**. In the case of the third-generation dendrimer **third-generation** **D8**, only one peak at 6.25 ppm referred to 168 protons for all aryl complexed groups in inert and outer spheres. As well, the NH groups of paracetamols appeared again at 10.13 ppm, and one Cp shifted upfield at 5.22 ppm referring to the 210 equivalent protons in both inner and outer rings which are surrounded by etheric oxygen groups at the para positions.

Successful synthesis of these dendrimers was also confirmed by using ^13^C NMR spectroscopy. For example, carbonyl groups showed one peak around 170.00 ppm for the **first generation D1** and increased by increasing the number of generations. Similarly, one peak in the dendrimers **D2**, **D3**, **D5**, **D6**, **D8**, **D9**, **D11**, **and** **D12** corresponded to the Cp carbons around 79.00 ppm. While two distinct Cp carbon peaks resonated around 80.00 and 79.00 ppm, for dendrimers **D1**, **D4**, **D7**, and **D10** there was a very clear difference between Cp peaks coordinated to the aryl groups with chloro and hydroxyl groups. Furthermore, in the presence of the peripheral chloro groups, the complexed carbons vibrated around 87.50 and 76.50 ppm, while the uncomplexed carbons with ester linkages located at 77.50 and 75.50 ppm. For example, in the **first-generation** **D3** with equivalent ester linkages resonated at 174.00 ppm, the complexed carbons resonated at 74.85 and 74.48 ppm, and the Cp carbons appeared at 78.83 ppm. However, in dendrimer **second-generation** **D4** with non-equivalent linkages, the complexed carbons pointed up at 86.43 and 74.71 ppm, while Cps which had two non-equivalent environments led to the appearance of two peaks at 78.95 and 77.44 ppm. Uncomplexed carbons were located in the specific area around 130.00 ppm and quaternary carbons were detected around 172.80 ppm.

The ATR-FTIR absorption spectra showed the presence and characteristic bands of hydroxyl, amine, ester, and ether groups, respectively, around 3400, 3330, and 1220 cm^–1^. The elemental analysis further confirmed the dendrimers’ formation as outlined in the experimental section. The solubility of the dendrimers in organic solvents decreased with increasing molecular weight. However, all of them were soluble in polar aprotic solvents such as DMF and DMSO.

**Figure 1:**
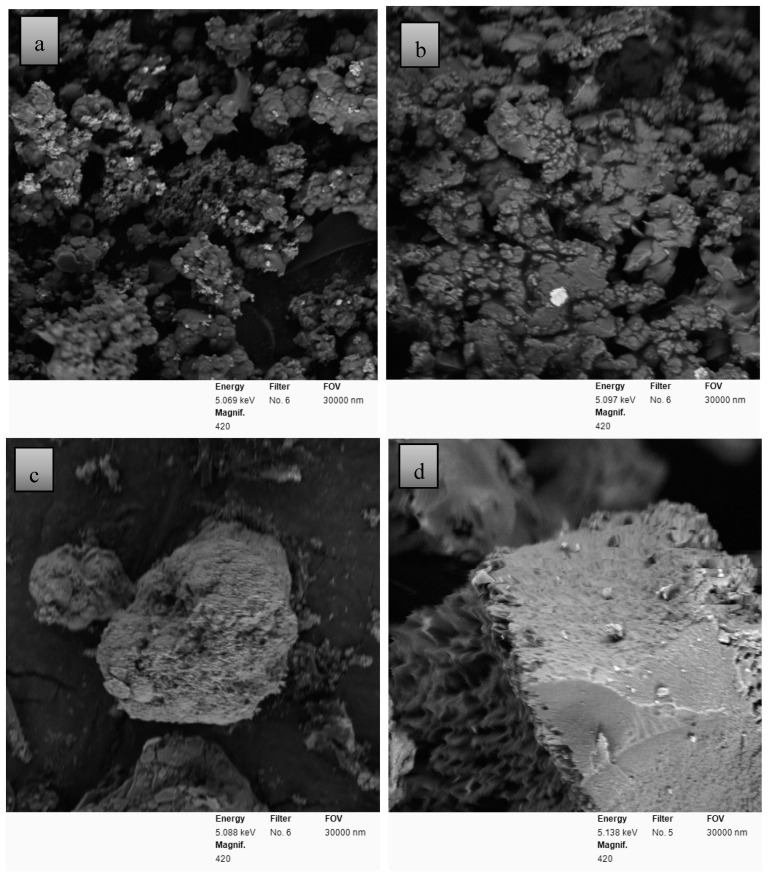
Scanning electron microscope (SEM) images (**a**) first-generation D2, (**b**) second-generation D5, (**c**) third-generation D8, (**d**) fourth-generation D11.

The morphology images of the dendrimers attached to paracetamol **first-generation D2**, **second-generation D5**, **third-generation D8**, and **fourth-generation D11** were taken by scanning electron microscopy (SEM), to show the difference between each generation. The microscopic images demonstrated the amorphous character of the dendrimers as shown in Figure 1.

As can be seen in Figure 1a, the first-generation D2 showed particles of almost the same size and shape arranged in a flower-like morphology with relatively uniform particle distribution. The image for the **second-generation** **D5** indicates an irregular shape with a slight aggregation of different sizes and shapes, with both large and small particles appearing with spaces between them. Although both first and second-generation had almost the same morphology, the **second-generation** **D5** composite was slightly larger than **first-generation D2**, as presented in Figure 1b. A sponge-like shape was shown in the **third-generation D8** and appeared as an irregular globule with many holes inside it as seen in Figure 1c. A rough surface was observed with large size in the **fourth-generation D11**, which contains ninety iron moieties, resulting in the particles having a rock-like appearance with sharp edges Figure 1d.

### 2.2. Electrochemical Properties

The redox properties of the dendrimers were studied using the cyclic voltammetry technique. η^6^-aryl-η^5^-cyclopentadienyliron(II) is redox-active in both the molecular and macromolecular scale and, therefore, the electrochemistry of these dendrimers features several remarkable trends [52,53,54]. The synthetic procedure allowed the including of redox-active iron centers η^6^-aryl-η^5^-cyclopentadienyliron (II) in the dendritic arms at every repeated synthetic step to form spheres of redox centers and the number of the redox centers increased from 6 units in the first-generation to 90 units in the fourth generation. The experiments were performed at temperatures of 0 °C in a solution of 0.1 M Bu_4_NPF_6_ using propylene carbonate as a supporting electrolyte, an Ag/Ag^+^ reference electrode, a glassy carbon working electrode, and a Pt wire counter electrode. The potential was scanned in the range of 0 to –2.0 V. All dendrimers exhibited a single redox wave with cathodic peak (E_pc_) and anodic peak (E_pa_) values, with changes in their intensities dependent on dendrimer generation.

**Figure 2:**
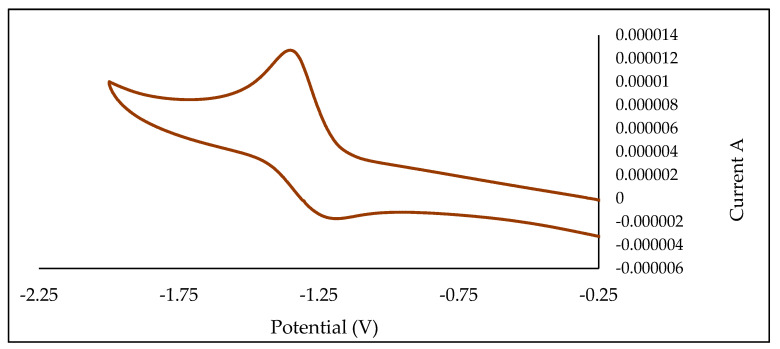
Representative cyclic voltammogram of **second-generation D6**, in 0.1 M Bu_4_NPF_6_ in propylene carbonate, scan rate = 0. 2 V/s, at −15 °C.

At room temperature dendrimers exhibited an irreversible redox process, while at 0 °C all dendrimers underwent reversible redox process, (Figure 2). The average *E*_1/2_ values were between –1.22 and –1.25 V, for first-generation dendrimers and the single redox was reasonable due to the equivalence of these redox centers. Dendrimers in the second-generation had a more negative shift and there was an increase in intensities in both reduction and oxidation peaks with the broadening of the peaks, as previously reported [55]. This shift was due to the higher number of cationic irons in this generation and the average *E*_1/2_ values were around –1.26 V.

The average *E*_1/2_ values were recorded to be up to –1.35 V in the fourth generation, a more negative shift appeared due to the increase of the number of redox-active centers, thus, the increase in their redox wave intensities. Also, the difference in the *E*_1/2_ values from first to the fourth generation was around –0.13 V as can be seen in Table 1 due to the greater number of cationic centers.

A split of the reverse peak was expected due to the different layers of the cationic redox dendrimers with different generations, but the high rate of electron transfer between the electrodes and iron centers restricted the splitting of the redox waves. Also, overlapped cathodic currents appeared from the reduction of iron centers consonant with previous studies [33].

### 2.3. Thermal Analysis

**Figure 3:**
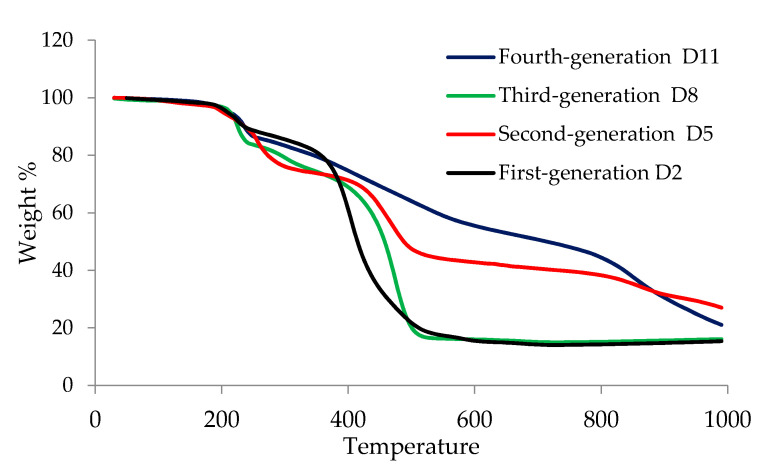
Thermogravimetric analysis (TGA) of generation first-generation D2, second-generation D5, third-generation D8, and fourth-generation D11.

The thermal stability of the dendrimers was investigated using TGA. The experiments were performed at atmospheric pressure under a nitrogen atmosphere. The samples’ weight loss as functions of temperature were recorded continuously, in the range of 0–1000 °C. The thermal decomposition of all dendrimers was begun around 190 °C, followed by a major weight loss in the temperature range of 350 to 600 °C. Some dendrimers displayed a third degradation step with minor weight losses above 850 °C, as shown in Figure 3 and Table 2.

The first decomposition is due to the losses of cationic cyclopentadienyl iron in the dendrimers’ arms, which exhibited around 15–25% loss at the temperature between 190–250 °C for as the first degradation of all dendrimers [31,56,57].

Each generation has almost the same degradation steps, for example, in the **first–generation D2**, the first loss was at 200–220 °C with 15%. The thermogram also showed a second massive weight loss accounting for 75% of its weight beginning at 380 °C and continues to decompose until beyond 580 °C, with 10% remaining at the end which referred to the iron residue. A broad range was noticed for the **second–generation D5** in its first degradation, with about 25% loss at a temperature between 190–250 ºC. In addition, two more degradation steps occurred, one at the range of 400 °C to 500 °C with 35% loss and the other one at 800 °C to 990 °C with only 10% loss, leaving 30% as iron residue. The **third–generation D8** with 42 iron centers had 20% loss between 200–210 °C, and also showed one main degradation step, with 70% loss beginning at 300 °C and ending at 500°C. The residual contents in this generation were found to be around 10%. Lastly, a loss of 20% was measured between 210–230 °C for the first degradation of the **fourth–generation D11** with 90 iron centers. Another smooth decomposition of the remaining complex was observed directly after the first degradation and ended after 1000 °C, with more than 70% loss.

### 2.4. Microbial Activity of Synthesized Dendrimers

The dendrimers were assayed against a broad spectrum of pathogenic microbes that included Gram–positive bacteria: methicillin–resistant *Staphylococcus aureus* (MRSA), vancomycin-resistant *Enterococcus faecium* (VRE), and *Staphylococcus warneri*; Gram-negative bacteria: *Pseudomonas aeruginosa* and *Proteus vulgaris;* and fungus *Candida albicans.* The concentrations at which inhibition of 50% of the growth was observed (IC_50_) and the minimum inhibitory concentration for inhibition of 90% of growth (MIC_90_), were determined. At the tested concentrations, all dendrimers were inactive against the Gram-negative bacteria and *C. albicans*. Most of the dendrimers were active against the Gram-positive bacteria with the minimum inhibitory concentration (MIC_90_) in the low micromolar range (Table 3).

The IC_50_ values showed in our study are equal or even lower than the antimicrobials in clinical use against MRSA and VRE. Dendrimers having chloro end groups exhibited notable antibacterial activity but not as much as the other end groups. We found dendrimers containing paracetamol groups in the outer spheres were more efficient against Gram-positive bacteria and slightly more efficacious compared to dendrimers carrying hydroxyl molecules in the periphery. It is likely that the presence of a terminal paracetamol group was responsible for the observed increase in efficacy in antimicrobial activity of their dendrimers (possibly due to target interactions with the N–H terminal group) [32,34,58]. This trend could show the action of primary amines against Gram-positive bacteria, which may also be related to the bacterial membrane permeability.

Recently, many studies have reported that the presence of the hydroxyl group at various positions of some compounds like flavonoids and coumarins enhanced their antibacterial activity [59,60,61,62]. In addition, the flexible short aliphatic chains attached to the hydroxyl group facilitates the interaction with the bacterial cytoplasmic membrane. The improvement of the antibacterial activity of different dendrimers with OH and NH_2_ end groups is due to the hindrance of bacterial biofilm production, bacterial cell membrane damage, and hindrance of virulence factors like enzymes and toxins [59]. The polarity of the NH_2_, OH group and the opportunity of intramolecular hydrogen bond formation could further role in these dendrimers and influence their antibacterial efficacy, comparable to the mechanism of action as rifampicin, which is a powerful antibiotic against mycobacterial infections as well as a broad range of Gram-positive and Gram-negative bacteria [63]. The geometry of these dendrimers and the chance of formation of the stable zwitterions by intermolecular hydrogen bonds provide high antimicrobial activity [63]. By dissolving rifampicin in water, the antibacterial ability was increased by both intra and intermolecular hydrogen bonding with water molecules which gave an additional stabilization on its zwitterionic form [63]. Our dendrimers with OH and NH_2_ end groups can form intra and intermolecular hydrogen bonding with DMSO molecules which gave them the minimum inhibitor activities than the other dendrimers by supporting the zwitterion form.

The activity of our dendrimers is also due to the production of reactive oxygen species (ROS) through electron transfer to oxygen molecules due to the presence of the redox-active Cp–Fe^II^–arene complex, as before confirmed [64,65]. The presence of ROS influences oxidative strain, a cellular protection technique employed against a broad spectrum of microbes [65,66,67].

Consequently, the presence of η^6^–arene–η^5^–cyclopentadienyliron(II) complex provided a further explanation for the excellent antibacterial activity against a wide spectrum of pathogenic microbes, particularly in the highest generation, due to the increase of the number of the η^6^–arene–η^5^–cyclopentadienyliron(II) complexes [33,43]. It is useful to mention that a single molecule of Cp–Fe^II^–arene complex reported an IC_50_ of 28.9 μM for MRSA, no activity has been shown for VRE, and 38.8 μM for *Staphylococcus warneri* [43], which demonstrated that the Cp–Fe^II^–arene complexes were themselves active against some types of Gram-positive bacteria, as previously reported [43]. Increasing the number of the redox–active Cp–Fe^II^–arene complexes increases the action of Cp–Fe^II^–arene on the pathogenic microbes by enhancing the interaction with the cell membrane and induction of oxidative stress on bacteria [33,34,43].

For example, for the **second–generation D4** (ending with chloro groups), an IC_50_ of 2.99 μM was observed for MRSA, 3.03 μM for VRE and 2.21 μM for *Staphylococcus warneri*. For the **second–generation D6** (ending with terminal hydroxyl groups) an IC_50_ of 2.70 μM was reported for MRSA, 2.64 μM for VRE, and 1.74 μM for *Staphylococcus warneri.* In the case of the **second-generation D5** (which ended in terminal paracetamol groups) an IC_50_ of 1.11 μM was observed for MRSA, 2.15 μM was recorded for VRE, and 1.14 μM for *Staphylococcus warneri*. Additionally, an increase in antibacterial activity was also recorded from first-generation dendrimers compared to higher generations. For example, for dendrimers having terminal paracetamol in the periphery, the **first-generation D2** exhibited an IC_50_ of 2.32 μM for MRSA, 4.19 μM for VRE, and 2.51 μM for *Staphylococcus warneri*. The **second-generation D5** displayed IC_50_ 1.11 μM for MRSA, 2.15 μM for VRE, and 1.14 μM for *Staphylococcus warneri,* while the **third-generation D8** showed an IC_50_ of 0.65 μM for MRSA, 1.69 μM for VRE, and 0.86 μM for *Staphylococcus warneri*. Excellent results were achieved with the **fourth-generation D11**, which presented IC_50_ of 0.53 μM for MRSA, 1.02 μM for VRE, and 0.73 μM for *Staphylococcus warneri.* A plausible explanation for this effect is due to the increase in the total surface area of the dendrimer periphery, increasing the number of available functionalized terminal groups that can interfere with the antibacterial target. Of the dendrimers tested, **fourth-generation D11** showed the most potent antibacterial activity against Gram-positive bacteria with efficacies better than the antimicrobial controls, vancomycin, and rifampicin (Table 3).

Compared with commercial paracetamol, the biological activity tests showed that the synthesized dendrimers **D2**, **D5**, **D8**, and **D11** with paracetamol moieties have higher antibacterial activity against these three Gram-positive bacteria in addition to the central analgesic effect. The inhibition was enhanced by increasing the number of η^6^-arene–η^5^-cyclopentadienyliron(II) complex from 6, 18, 42, and 90 in the dendritic branches.

## 3. Experimental

### 3.1. Materials

All chemicals and reagents were purchased from Sigma-Aldrich, Oakville, Ontario, Canada and were used without any further purification. All solvents were dried and stored over 3 Å molecular sieves before being used. The synthesis of the organoiron complex 1 followed previously reported procedures [68,69].

### 3.2. Instrumentation

A Bruker Avance nuclear magnetic resonance (NMR) spectrometer (^1^H, 300 MHz and ^13^C, 75 MHz), Billerica, Middlesex County, Massachusetts, US, was used to characterize all synthesized complexes in DMSO-d_6_ with the chemical signals referenced to solvent residual signal in ppm. Attenuated total reflection Fourier transform IR (ATR-FTIR) absorption spectroscopic measurements were acquired on a Bruker Alpha FTIR spectrometer Alpha-P, Billerica, Middlesex County, Massachusetts, US. Cyclic voltammetric experiments were carried out on a Princeton Applied Research/EG&G Model 263 potentiostat/galvanostat, champaign, USA. Using glassy carbon working electrode, Pt counter electrode, and Ag reference electrode. The experiments, which were carried out at a scan rate between 0.1 and 1.5 Vs^–1^ at 0 °C under nitrogen atmosphere in degassed propylene carbonate as solvent and tetrabutylammonium hexafluorophosphate as supporting electrolyte. Scanning electron micrographs were obtained on an LVEM5 benchtop instrument which operates at 5 kV, Delong America, Montreal Quebec Canada. Powdered samples were cast onto stubs, dried under vacuum, and coated with gold/palladium before imaging. TGA was conducted in platinum pans under nitrogen at a heating rate of 10 °C on a TA Instruments TGA Q500 Mississauga, Canada.

### 3.3. Antimicrobial Assay

All microbroth antibiotic susceptibility testing was carried out according to Overy et al. [70] using the following pathogens: methicillin-resistant *Staphylococcus aureus* ATCC 33591 (MRSA); *Staphylococcus warneri* ATCC 17917, vancomycin-resistant *Enterococcus faecium* EF379 (VRE); *Pseudomonas aeruginosa* ATCC 14210, *Proteus vulgaris* ATCC 12454, and *Candida albicans* ATCC 14035. Complexes were serially diluted to generate a range of eight concentrations (128 µg/mL to 1 µg/mL) in a final well volume concentration of 2% DMSO (aq). Each plate contained three uninoculated positive controls, three untreated negative controls, and an appropriate concentration range of a control antibiotic (vancomycin for MRSA and *Staphylococcus warneri*, rifampicin for VRE, gentamycin for *P. aeruginosa*, ciprofloxacin for *P. vulgaris*, and nystatin for *C. albicans*). The optical density of the plate was recorded using a Thermo Scientific Varioskan Flash plate reader at 600 nm at time zero and then again after incubation of the plates for 22 h at 37 °C. After subtracting the time zero OD_600_ from the final reading, the percentages of microorganism survival relative to vehicle control wells were calculated.

## 4. Synthesis and Characterization

Four generations of redox-active organoiron dendrimers with three different end groups: chloro, hydroxyl, and paracetamol-terminated dendrimers were synthesized by using esterification reaction and the nucleophilic aromatic substitution (S_N_Ar) reaction. These dendrimers had a different number of the end groups starting from 6 for the first-generation up to 48 in the fourth generation. Also, the dendrimers’ branches contained between 6 to 90 units of the redox-active complex η^6^-aryl-η^5^-cyclopentadienyliron (II), which drove the reaction to occur in a moderated condition as well as improving the antibacterial efficiency of the dendrimers. Functionalization of the dendrimers with paracetamol yielded hybrid antimicrobial dendrimers with enhanced activity, especially at higher generation.

### 4.1. General Procedure

The core, 1, 3, 5-trihydroxybenzene (Phloroglucinol), and complex **1** were used to build first-generation dendrimer **D1** (**first-generation D1**), by using Steglich esterification [69] with a molar ratio of 1:3. The solutions were stirred at 0 °C under nitrogen atmosphere for 15 min. Then the closed system was stirred at room temperature for two days. The reaction mixture was cooled to −25 °C in a freezer for three hours, filtered to remove dicyclohexylurea (DHU) and then poured into 10% HCl solution. The precipitate was filtrated and then dissolved in acetone, cooled again to −25 °C in a freezer for another three hours, filtered to remove any extra remaining DHU, and removal of the solvent by evaporation or reprecipitation in 10% HCl solution gave rise to the products. The same methodology was used to synthesize higher generations dendrimers **D4**, **D7**, and **D10**, by using 1:6, 1:12, and 1:24 molar ratios, respectively.

Nucleophilic aromatic substitution reactions were used in the synthesis of dendrimer **D2**, **D5**, **D8**, and **D11** by using 1:6, 1:12, 1:24, and 1:48 molar ratios of the core to paracetamol. The closed reaction mixtures were stirred in DMF and K_2_CO_3_ at room temperature between two to three days after flushing with nitrogen for 30 min. Subsequently, the reaction mixtures were poured into a 10% HCl solution, and NH_4_PF_6_ was added to precipitate the products. The products were collected by suction filtration and dried under vacuum at room temperature. The same method was used in the synthesis of dendrimer **D3**, **D6**, **D9**, and **D12**, by using 4-hydroxybenzyl alcohol instead of paracetamol with 1:6, 1:12, 1:24, and 1:48 molar ratios, respectively. The detailed synthetic methodologies for the dendrimers and their precursors, and spectroscopic characterization including ^1^H and ^13^C NMR, ATR-FTIR, and elemental analyses are reported below.

### 4.2. Synthesis and Characterization of Dendrimers D1–D12

#### 4.2.1. Chloro-Terminal Dendrimer (First-Generation D1)

A 25 mL round-bottom flask was charged with bimetallic organoiron complex **1** (0.50 g, 0.48 mmol), tri-hydroxybenzene (0.02 g, 0.18 mmol), and 4-(Dimethylamino)pyridine (DMAP) (0.11g, 1.80 mmol), and dissolved in 10 mL of DMF. The solution was stirred in an ice bath under a nitrogen atmosphere while N,N′-Dicyclohexylcarbodiimide (DCC) (0.09 g, 0.48 mmol) was added over a 15-min period. The reaction mixture was stirred under nitrogen for 24 h. The product was poured into 100 mL of 10% HCl solution, and NH_4_PF_6_ (0.15 g, 1.9 mmol) was added to generate a precipitate.

Molecular weight 3190 g/mol and yield 78%. ATR-FTIR; *ν_max_*/cm^–1^: 2997 (Ar–C), 2876 (Cp–C), 1698 (CO), 1223 (C–O–C). ^1^H NMR data δ_H_ (300 MHz; DMSO-*d_6_*): 7.41 (12H, t, *J = 8.4 Hz*, uncomplexed Ar–H), 7.35 (12H, d, *J* = 6.9 Hz, uncomplexed Ar–H), 7.11 (3H, s, uncomplexed Ar–H), 6.82 (12H, d, *J* = 6.0 Hz, complexed Ar–H), 6.43 (12H, d, *J* = 6.0 Hz, complexed Ar–H), 5.29 (30H, s, Cp–H), 2.41 (6H, s, CH_2_), 2.09 (6H, s, CH_2_), 1.73 (9H, s, CH_3_). ^13^C NMR *δ*_c_ (75 MHz; DMSO-*d_6_*): 152.17 (CO), 146.77, 132.85, and 104.57 (quat-C), 132.86, 130.19, 121.14, 107.64, and 100.94 (uncomplexed Ar–C), 80.27 (Cp–C), 87.71 and 77.30 (complexed Ar–C), 31.01 and 25.85 (CH_2_–C), 28.31 (CH_3_–C). The elemental analysis of C_123_H_102_O_12_Cl_6_Fe_6_P_6_F_36_: calculated: %C 46.32, %H 3.22, and found %C 46.84 and %H 2.99.

#### 4.2.2. Paracetamol-Terminal Dendrimer (First-Generation D2)

Dendrimer **D2** was synthesized through a nucleophilic aromatic substitution reaction using dendrimer **D1** and acetaminophen **2**. A 25 mL round-bottom flask was charged with acetaminophen (0.03 g, 0.18 mmol), dendrimer **D1** (0.10 g, 0.03 mmol), and K_2_CO_3_ (0.13 g, 0.94 mmol), in 7 mL of DMF. The closed reaction mixture was stirred at room temperature for two days after flushing with nitrogen for 1 h. Subsequently, the reaction mixture was poured into 100 mL of HCl solution, and NH_4_PF_6_ (0.02 g, 0.24 mmol) was added to precipitate the product. The product was filtered and dried under vacuum resulting in a yellowish solid product.

Molecular weight 3878 g/mol and yield 82%. ATR-FTIR; *ν_max_*/cm^–1^: 3396 (NH), 2956 (Ar–C), 2913 (Cp–C), 1714 (CO), 1243 (C–O–C). ^1^H NMR data δ_H_ (300 MHz; DMSO-*d_6_*): 10.11 (6H, s, NH), 7.34 (12H, d, *J* = 8.4 Hz*,* uncomplexed Ar–H), 7.56 (24H, d, *J* = 9.0 Hz, uncomplexed Ar–H), 7.23 (12H, d, *J* = 8.7 Hz uncomplexed Ar–H), 7.01 (3H, s, uncomplexed Ar–H), 6.22 (24H, d, *J* = 7.2 Hz, complexed Ar–H), 5.20 (30H, s, Cp–H), 2.37 (6H, s, CH_2_), 2.11 (6H, s, CH_2_), 2.01 (18H, s, CH_3_), 1.65 (9H, s, CH_3_). ^13^C NMR *δ*_c_ (75 MHz; DMSO-*d_6_*): 169.26, 152.62 (CO), 148.78, 144.53, 138.46, 131.74, and 130.68 (quat-C), 130.069, 122.69, 122.08, 121.67, 120.76, and 100.90 (uncomplexed Ar–C), 78.69 (Cp–C), 76.01 and 75.05 (complexed Ar–C), 32.42 and 30.65 (CH_2_–C), 27.87 and 25.13 (CH_3_–C). The elemental analysis of C_171_H_150_O_24_N_6_Fe_6_P_6_F_36_: calculated %C 52.96, %H 3.90, %N 2.17, and found %C 53.68, %H 4.13, and %N 2.47.

#### 4.2.3. Hydroxyl-Terminal Dendrimer (First-Generation D3)

Dendrimer **D3** was synthesized from dendrimer **D1** (0.20 g, 0.06 mmol), 4-hydroxybenzyl alcohol **3** (0.05 g, 0.30 mmol), and K_2_CO_3_ (0.26 g, 1.80 mmol) in 7 mL of DMF through nucleophilic substitution reaction. The reaction was left to stir at room temperature for three days after flushing with nitrogen for 1 h. Subsequently, the reaction mixture was poured into 100 mL of 10% HCl and NH_4_PF_6_ (0.02 g, 0.24 mmol) was added for complete precipitation. The product was filtered and dried under vacuum. The resulting yellow solid was collected by suction filtration and dried under vacuum at room temperature.

Molecular weight 3716 g/mol and yield 66%. ATR-FTIR; *ν_max_*/cm^–1^: 3338 (OH), 2956 (Ar–C), 2913 (Cp–C), 1714 (CO), 1243 (C–O–C). ^1^H NMR data δ_H_ (300 MHz; DMSO-*d_6_*): 7.48 (12H, d, *J* = 8.4 Hz*,* uncomplexed Ar–H), 7.37–7.23 (36H, m, uncomplexed Ar–H), 7.04 (3H, s, uncomplexed Ar–H), 6.25 (24H, d, *J* = 6.0 Hz, complexed Ar–H), 5.32 (6H, d, *J* = 5.4 Hz*,* OH), 5.22 (30H, s, Cp–H), 4.55 (12H, d, *J = 6.3 Hz*, CH_2_), 2.40 (6H, s, CH_2_), 2.12 (6H, s, CH_2_), 1.66 (9H, s, CH_3_). ^13^C NMR *δ*_c_ (75 MHz; DMSO-*d_6_*): 174 (CO), 162.04, 159.62, 156.48, 153.21, 145.67, 140.48, and 133.85 (quat-C), 130.09, 129.97, 129.83, 129.57, 127.98, 121.21, and 120.75 (uncomplexed Ar–C), 78.83 (Cp–C), 74.85 and 74.48 (complexed Ar–C), 45.06, 26.33, and 25.99 (CH_2_–C), 30.40 (CH_3_–C). The elemental analysis of C_165_H_144_O_24_Fe_6_P_6_F_36_: calculated %C 53.33, %H 3.98, and found %C 53.86, and %H 4.20.

#### 4.2.4. Chloro-Terminal Dendrimer (Second-Generation D4)

In a procedure analogous to the synthesis of **D1**, dendrimer **D4** was synthesized from dendrimer **D3** (0.16 g, 0.29 mmol), bimetallic organoiron complex **1** (0.27 g, 1.50 × 10^4^ mmol), DMAP (0.06 g, 0.50 mmol), and DCC (0.05 g, 0.25 mmol) in 7 mL DMF. The resulting yellow solid was collected by suction filtration and dried under vacuum at room temperature.

Molecular weight 9843 g/mol and yield 62%. ATR-FTIR; *ν_max_*/cm^–1^: 3002 (Ar–C), 2935 (Cp–C), 1721 (CO), 1230 (C–O–C). ^1^H NMR data δ_H_ (300 MHz; DMSO-*d_6_*): 7.37 (48H, t, *J* = 8.5 Hz, uncomplexed Ar–H), 7.29 (48H, d, *J* = 8.7 Hz, uncomplexed Ar–H), 7.02 (3H, s, uncomplexed Ar–H), 6.82 (24H, d, *J* = 6.0 Hz, complexed Ar–H), 6.43 (24H, d, *J* = 6.0 Hz, complexed Ar–H), 6.26 (24H, d, *J* = 6.0 Hz, complexed Ar–H), 5.28 (60H, s, Cp–H), 5.22 (30H, s, Cp–H), 4.55 (12H, d, *J* = 5.4 Hz*,* CH_2_), 2.41 (18H, s, CH_2_), 2.09 (18H, s, CH_2_), 1.68 (18H, s, CH_3_) 1.62 (9H, s, CH_3_). ^13^C NMR *δ*_c_ (75 MHz; DMSO-*d_6_*): 173.97 and 171.32 (CO), 152, 146.77, 132.85, and 103.25 (quat-C), 133.63, 132.95, 131.76, 131.53, 129.46, 128.84, 128.20, 126.18, 119.77, 119.49, 117.99, and 103.25 (uncomplexed Ar–C), 78.95 and 77.44 (Cp–C), 86.43 and 74.71 (complexed Ar–C), 61.73, 52.14, and 24.91 (CH_2_–C), 31.14 and 24.91 (CH_3_–C). The elemental analysis of C_399_H_336_O_42_Cl_12_Fe_18_P_18_F_108_: calculated %C 48.69, %H 3.44, and found %C 49.15, and %H 3.66.

#### 4.2.5. Paracetamol-Terminal Dendrimer (Second-Generation D5)

In a procedure analogous to the synthesis of **D2**, dendrimer **D5** was synthesized from dendrimer **D4** (0.20 g, 0.02 mmol), acetaminophen **2** (0.03 g, 0.24 mmol), and K_2_CO_3_ (0.17 g, 1.20 mmol), in 7 mL DMF. The product was a yellow powder.

Molecular weight 11,219 g/mol and yield 53%. ATR-FTIR; *ν_max_*/cm^–1^: 3401 (NH), 2877 (Ar–C), 2823 (Cp–C), 1703 (CO), 1223 (C–O–C). ^1^H NMR data δ_H_ (300 MHz; DMSO-*d_6_*): 10.13 (12H, s, NH), 7.75 (24H, d, *J* = 7.2 Hz*,* uncomplexed Ar-H), 7.34 (60H, d, *J* = 7.2 Hz, uncomplexed Ar–H), 7.25 (60H, d, *J* = 9.0 Hz, uncomplexed Ar–H), 7.00 (3H, s, uncomplexed Ar–H), 6.25 (72H, d, *J* = 6.6 Hz, complexed Ar–H), 5.22 (90H, s, Cp–H), 4.56 (12H, d, *J* = 6 Hz*,* CH_2_), 2.41 (18H, s, CH_2_), 2.03 (18H, s, CH_2_), 1.67 (36H, s, CH_3_), 1.65 (18H, s, CH_3_), 1.61 (9H, s, CH_3_). ^13^C NMR *δ*_c_ (75 MHz; DMSO-*d_6_*): 174.48 and 168.44 (CO), 151.76, 147.98, 146.18, 140.81, 137.69, 130.87, and 129.19 (quat-C), 129.19, 128.65, 121.21, 120.85, 120.37, and 119,92 (uncomplexed Ar–C), 77.91 (Cp–C), 76.32 and 74.24 (complexed Ar–C),62.18, 33.34, 31.63, and 24.46 (CH_2_–C), 25.39 and 24.01 (CH_3_–C). The elemental analysis of C_495_H_432_O_24_N_12_Fe_18_P_18_F_108_: calculated %C 52.99, %H 3.88, %N 1.50, and found %C 53.51, %H 4.19, and %N 1.62.

#### 4.2.6. Hydroxyl-Terminal Dendrimer (Second-Generation D6)

In a procedure analogous to the synthesis of **D3**, dendrimer **D6** was synthesized from dendrimer **D4** (0.20 g, 0.02 mmol), 4-hydroxybenzyl alcohol **3** (0.03 g, 0.26 mmol), and K_2_CO_3_ (0.18 g, 1.30 mmol), in 7 mL DMF.

Molecular weight 10,895 g/mol and yield 52%, yellow powder. ATR-FTIR; *ν_max_*/cm^–1^: 3334 (OH), 2944 (Ar–C), 2962 (Cp–C), 1694 (CO), 1213 (C–O–C). ^1^H NMR data δ_H_ (300 MHz; DMSO-*d_6_*): 7.48 (36H, d, *J* = 8.1 Hz*,* uncomplexed Ar–H), 7.32–7.21 (108H, m, uncomplexed Ar–H), 7.01 (3H, s, uncomplexed Ar–H), 6.22 (72H, s, complexed Ar–H), 5.33 (12H, d, *J* = 5.4 Hz*,* OH), 5.20 (90H, s, Cp–H), 4.53 (36H, d, *J* = 5.4 Hz, CH_2_), 2.38 (18H, s, CH_2_), 2.04 (18H, s, CH_2_), 1.64 (9H, s, CH_3_), 1.59 (18H, s, CH_3_). ^13^C NMR *δ*_c_ (75 MHz; DMSO-*d_6_*): 174.11, 172.22, and 162.04 (CO), 154.084, 152.63, 141.65, 140.75, 140.39, 140.10, 139.52, 138.65, and 133.04 (quat-C), 131.32, 130.91, 130.30, 130.05, 129.97, 129.81, 129.54, 129.45, 129.05, 128.08, 127.96, 121.23, 120.78, 118.87, and 118.41 (uncomplexed Ar–C), 78.84 (Cp–C), 75.97 and 75.48 (complexed Ar–C), 65.79, 63.05, 43.89, 26.27, and 25.27 (CH_2_–C), 30.42, 23.03, and 19.33 (CH_3_–C). The elemental analysis of C_483_H_420_O_66_Fe_18_P_18_F_108_: calculated %C 53.25, %H 3.89, and found %C 53.71, and %H 4.06.

#### 4.2.7. Choro-Terminal Dendrimer (Third-Generation D7)

In a procedure analogous to the synthesis of **D1**, dendrimer **D7** was synthesized from dendrimer **D6** (0.10 g, 9.20 µmol), bimetallic organoiron complex **1** (0.12 g, 12 µmol), DMAP (0.03 g, 18.40 µmol), and DCC (0.03 g, 9.20 µmol) in 7 mL DMF. The resulting yellow powder was collected by suction filtration and dried under vacuum at room temperature.

Molecular weight 23,149 g/mol and yield 44%. ATR-FTIR; *ν_max_*/cm^–1^: 3021 (Ar–C), 2889 (Cp–C), 1692 (CO), 1237 (C–O–C). ^1^H NMR data δ_H_ (300 MHz; DMSO-*d_6_*): 7.48 (24H, d, *J* = 8.4 Hz*,* uncomplexed Ar–H), 7.32 (96H, s, uncomplexed Ar–H), 7.29 (96H, d, *J* = 8.7 Hz, uncomplexed Ar–H), 7.02 (27H, s, uncomplexed Ar–H), 6.81 (48H, d, *J* = 6.6 Hz, complexed Ar–H), 6.40 (48H, s, complexed Ar–H), 6.26 (72H, d, *J* = 7.8 Hz, complexed Ar–H), 5.28 (120H, s, Cp–H), 5.22 (90H, s, Cp–H), 4.55 (36H, s*,* CH_2_), 2.39 (42H, s, CH_2_), 2.07 (42H, s, CH_2_), 1.64 (63H, br–s, CH_3_). ^13^C NMR *δ*_c_ (75 MHz; DMSO-*d_6_*): 175.26, 173.59, and 170.21 (CO), 154.28, 152.65, 152.03, 147.27, 147.06, 141.64 133.00, 131.40, and 104.53 (quat-C), 130.82, 130.189, 121.35, 121.161, 121.01, and 120.78 (uncomplexed Ar–C), 80.19 and 78.81 (Cp–C), 87.69, 77.32, and 76.05 (complexed Ar–C), 65.95, 63.14, 45.97, 34.27, and 31.18 (CH_2_–C), 36.65, 31.68, and 27.76 (CH_3_–C). The elemental analysis of C_951_H_804_O_42_Cl_24_Fe_42_P_42_F_252_: Calculated %C 49.34, %H 3.50, and found %C 50.23 and %H 3.69.

#### 4.2.8. Paracetamol-Terminal Dendrimer (Third-Generation D8)

In a procedure analogous to the synthesis of **D2**, dendrimer **D8** was synthesized from dendrimer **D7** (0.10 g, 4.18 µmol), acetaminophen **2** (0.02 g, 108.00 µmol), and K_2_CO_3_ (0.10 g, 0.50 mmol), in 7 mL DMF.

Molecular weight 25,902 g/mol and yield 61% as yellow powder. ATR-FTIR; *ν_max_*/cm^–1^: 3437 (NH), 2932 (Ar–C), 2823 (Cp–C), 1708 (CO), 1217 (C–O–C). ^1^H NMR data δ_H_ (300 MHz; DMSO-*d_6_*): 10.13 (24H, s, NH), 7.76 (36H, d, *J* = 8.7 Hz*,* uncomplexed Ar–H), 7.34 (168H, s, uncomplexed Ar–H), 7.25 (84H, d, *J* = 9.3 Hz, uncomplexed Ar–H), 7.04 (51H, s, uncomplexed Ar–H), 6.25 (168H, d, *J* = 7.2 Hz, complexed Ar–H), 5.22 (210H, s, Cp–H), 5.06 (24H, s, CH_2_), 4.55 (12H, s, CH_2_), 2.41 (42H, s, CH_2_), 2.03 (42H, s, CH_2_), 1.65 (135H, s, CH_3_). ^13^C NMR *δ*_c_ (75 MHz; DMSO-*d_6_*): 175, 173.62, 171.65, and 169.32 (CO), 153.50, 148.99, 138.51, 131.74, 131.33, 130.65, and 129.46 (quat-C), 130.04, 122.04, 121.707, 121.35, 121.18, 120.72, and 119,82 (uncomplexed Ar–C), 78.69 (Cp–C), 76.04 and 75.03 (complexed Ar–C), 65.87, 63.02, 45.78, 34.25, 30.84, and 25.35 (CH_2_–C), 49.03, 27.57, and 24.80 (CH_3_–C). The elemental analysis of C_1143_H_996_O_150_N_24_Fe_42_P_42_F_252_: calculated %C 53.00, %H 3.88, %N 1.30, and found %C 53.76, %H 4.11, and %N 1.51.

#### 4.2.9. Hydroxyl-Terminal Dendrimer (Third-Generation D9)

In a procedure analogous to the synthesis of **D3**, dendrimer **D9** was synthesized from dendrimer **D7** (0.10 g, 4.18 µmol), 4-hydroxybenzyl alcohol **3** (0.01 g, 108.00 µmol), and K_2_CO_3_ (0.10 g, 0.50 mmol), in 7 mL DMF. The product was a yellow powder.

Molecular weight 25,254 g/mol and yield 58%. ATR-FTIR; *ν_max_*/cm^–1^: 3321 (OH), 2873 (Ar–C), 2897 (Cp–C), 1722 (CO), 1221(C–O–C). ^1^H NMR data δ_H_ (300 MHz; DMSO-*d_6_*): 7.47 (168H, d, *J* = 7.2 Hz*,* uncomplexed Ar–H), 7.26 (84H, m, uncomplexed Ar–H), 7.00 (87H, s, uncomplexed Ar–H), 6.26 (168H, s, complexed Ar–H), 5.29 (24H, s, OH), 5.20 (210H, s, Cp–H), 4.53 (48H, s, CH_2_), 2.36 (36H, s, CH_2_), 2.16 (42H, s, CH_2_), 2.05 (42H, s, CH_2_), 1.88 (9H, s, CH_3_), 1.63 (18H, s, CH_3_), 1.59 (36H, s, CH_3_). ^13^C NMR *δ*_c_ (75 MHz; DMSO-*d_6_*): 174.11, 172.22, and 162.04 (CO), 154.084, 152.63, 141.65, 140.75, 140.39, 140.10, 139.52, 138.65, and 133.04 (quat-C), 131.32, 130.91, 130.30, 130.05, 129.97, 129.81, 129.54, 129.45, 129.05, 128.08, 127.96, 121.23, 120.78, 118.87, and 118.41 (uncomplexed Ar–C), 78.84 (Cp–C), 75.97 and 75.48 (complexed Ar–C), 65.79, 63.05, 43.89, 26.27, and 25.27 (CH_2_–C),36.12, 31.42, 27.03, and 19.33 (CH_3_–C). The elemental analysis of C_1119_H_972_O_150_Fe_42_P_42_F_252_: calculated %C 53.22, %H 3.88, and found %C 54.37, and %H 4.03.

#### 4.2.10. Chloro Terminal Dendrimer (Fourth-Generation D10)

In a procedure analogous to the synthesis of **D1**, dendrimer **D10** was synthesized from dendrimer **D9** (0.20 g, 7.60 µmol), bimetallic organoiron complex (0.19 g, 190.00 µmol), DMAP (0.02 g, 380.00 µmol), and DCC (0.03 g, 190.00 µmol) in 7 mL DMF. The resulting yellow solid was collected by suction filtration and dried under vacuum at room temperature.

Molecular weight 49,762 g/mol and yield 71%. ATR-FTIR; *ν_max_*/cm^–1^: 2946 (Ar–C), 2847 (Cp–C), 1722 (CO), 1221 (C–O–C). ^1^H NMR data δ_H_ (300 MHz; DMSO-*d_6_*): 7.49 (264H, m, uncomplexed Ar–H), 7.37 (267H, m, uncomplexed Ar–H), 6.82 (96H, m, complexed Ar–H), 6.43 (96H, m, complexed Ar–H), 6.27 (168H, m, complexed Ar–H), 5.28 (240H, s, Cp–H), 5.22 (210H, s, Cp–H), 5.12 (21H, s, CH_2_), 4.56 (42H, s, CH_2_), 3.97 (21H, s, CH_2_), 2.41 (90H, br, s, CH_2_), 2.34 (90H, br, s, CH_2_), 1.68 (108H, m, CH_3_), 1.63 (27H, s, CH_3_). ^13^C NMR *δ*_c_ (75 MHz; DMSO-*d_6_*): 172.47 and 172.27 (CO), 154.32, 152.72, 151.99, 147.01, 141.70, 132.83, and 104.54 (quat–C), 131.34, 130.12, 129.51, 120.98, and 120.71 (uncomplexed Ar–C), 80.29 and 78.74 (Cp–C), 87.71, 77.30, 75.97, and 75.42 (complexed Ar–C), 65.84, 63.01, 32.50, 31.12, and 25.38 (CH_2_–C), 30.54 and 26.19 (CH_3_–C). The elemental analysis of C_2055_H_1740_O_222_Cl_48_Fe_90_P_90_F_540_: calculated %C 49.60.35, %H 3.52, and found %C 50.09 and %H 3.78.

#### 4.2.11. Paracetamol-Terminal Dendrimer (Fourth-Generation D11)

In the procedure analogous to the synthesis of **D2**, dendrimer **D11** was synthesized from dendrimer **D10** (0.10 g, 1.90 µmol), acetaminophen **2** (0.02 g, 96.00 µmol), and K_2_CO_3_ (0.10 g, 0.45 mmol), in 7 mL DMF. The product was a yellow powder.

Molecular weight 63,428 g/mol and yield 59%. ATR-FTIR; *ν_max_*/cm^–1^: 3464 (NH), 2944 (Ar–C), 2811 (Cp–C), 1716 (CO), 1231 (C–O–C). ^1^H NMR data δ_H_ (300 MHz; DMSO-*d_6_*): 10.13 (48H, s, NH), 7.76 (96H, d, *J* = 8.7 Hz*,* uncomplexed Ar–H), 7.49 (264H, m, uncomplexed Ar–H), 7.37 (363H, m, uncomplexed Ar–H), 6.27 (360H, m, complexed Ar–H), 5.22 (450H, s, Cp–H), 4.56 (42H, s, CH_2_), 3.97 (42H, s, CH_2_), 2.41 (90H, br, s, CH_2_), 2.34 (90H, br, s, CH_2_), 1.72 (144H, s, CH_3_), 1.65 (135H, s, CH_3_). ^13^C NMR *δ*_c_ (75 MHz; DMSO-*d_6_*): 175.31, 173.44, 171.21 and 169.43 (CO), 152.70, 148.84, 146.87, 141.62, 138.45, 131.76, 131.33, 130.72 and 129.21 (quat-C), 130.04, 122.04, 121.707, 121.35, 121.18, 120.72 and 119,82 (uncomplexed Ar–C), 78.74 (Cp–C), 75.97 and 75.05 (complexed Ar–C), 63.17, 45.75, 36.98, 32.42, 26.86 and 25.35 (CH_2_–C), 27.86 and 24.75 (CH_3_–C). The elemental analysis of C_2439_H_2124_O_318_N_48_Fe_90_P_90_F_540_: calculated %C 53.00, %H 3.87, %N 1.22, and found %C 53.67, %H 4.01, %N 1.51.

#### 4.2.12. Hydroxyl-Terminal Dendrimer (Fourth-Generation D12)

Similarly, the synthesis of **D3**, dendrimer **D12** was synthesized from dendrimer **D10** (0.10 g, 1.90 µmol), 4-hydroxybenzyl alcohol **3** (0.01 g, 96.00 µmol), and K_2_CO_3_ (0.07 g, 2.30 mmol), in 7 mL DMF. The product was a yellow solid.

Molecular weight 53,971 g/mol and yield 51%. ATR-FTIR; *ν_max_*/cm^–1^: 3324 (OH), 2972 (Ar–C), 2898 (Cp–C), 1714 (CO), 1222 (C–O–C).^1^H NMR data δ_H_ (300 MHz; DMSO-*d_6_*): 7.49 (96H, m, uncomplexed Ar–H), 7.47 (264H, m, uncomplexed Ar–H), 7.37 (267H, m, uncomplexed Ar–H), 7.03 (96H, m, uncomplexed Ar–H), 6.26 (360H, d, *J* = 6.0 Hz*,* complexed Ar–H), 5.29 (48H, s, OH), 5.22 (450H, s, Cp–H), 5.12 (21H, s, CH_2_), 4.55 (42H, s, CH_2_), 3.97 (21H, s, CH_2_), 2.41 (90H, br, s, CH_2_), 2.22 (96H, br s, CH_2_), 2.08 (90H, br, s, CH_2_), 1.66 (108H, m, CH_3_), 1.62 (27H, s, CH_3_).^13^C NMR *δ*_c_ (75 MHz; DMSO-*d_6_*): 175.26, 173.58, and 163.19 (CO), 163.19, 152.67, 152,51, 146.51, 141.63, 131.41, and 130.75 (quat-C), 130.05, 129.47, 121.19, 120.78 and 119.33 (uncomplexed Ar–C), 78.80 (Cp–C), 76.03 and 75.46 (complexed Ar–C), 63.13, 45.92, 37.65, 32.49, 31.64, 30.63, and 25.14 (CH_2_–C), 37.04 and 26.28 (CH_3_–C). The elemental analysis of C_2391_H_2076_O_318_Fe_90_P_90_F_540_: Calculated %C 53.21, %H 3.88, and found %C 52.83 and %H 4.11.

## 5. Conclusions

In summary, four generations of cationic, redox-active organometallic dendrimers were synthesized with three different end groups. The biologically active organoiron complex was used to synthesize the first paracetamol dendrimers with up to 90 redox-active units in the highest generation. All dendrimers were examined as antimicrobial agents against a broad spectrum of pathogenic microbes that included Gram-positive bacteria and Gram-negative bacteria. High levels of inhibition have been shown for the Gram-positive bacteria, while no activity was observed for the Gram-negative bacteria or *C. albicans*. The effectiveness of these antimicrobial agents against the Gram-positive bacteria were due to the organometallic dendritic scaffold and the terminal end groups. The effect on the Gram-positive bacteria may have occurred via two mechanisms: interaction with the cell membrane and the induction of oxidative stress on bacteria. The results suggested that both mechanisms participated in the antimicrobial activity of these dendrimers. Moreover, functionalization enhanced the antimicrobial impact on the dendrimers, with the secondary amine group-functionalized paracetamol dendrimers being more active than others. It is worth mentioning that increasing the number of generations of dendrimers profoundly influenced the activity. The best result has been recorded for the **fourth-generation D11**, which was attached to 48 paracetamol end groups and has 90 units of η^6^-aryl-η^5^-cyclopentadienyliron (II) complex. These macromolecules, functionalized with paracetamol moieties, obtained high potential activity against Gram-positive bacteria in addition to the central analgesic effect. In future work, we plan to create more bioactive dendrimers and test them against numerous types of infectious microbe as well as investigating them for potential use as anticancer agents.

## Figures and Tables

**Table 1:** Redox activity of dendrimers **1–12** at 0 °C.

Dendrimers	E_pc_ (V)	E_pa_ (V)	E_1/2_ (V)
**D1**	−1.26	−1.21	−1.24
**D2**	−1.26	−1.24	−1.25
**D3**	−1.28	−1.16	−1.22
**D4**	−1.32	−1.21	−1.27
**D5**	−1.28	−1.26	−1.27
**D6**	−1.29	−1.23	−1.26
**D7**	−1.32	−1.14	−1.23
**D8**	−1.32	−1.36	−1.34
**D9**	−1.33	−1.14	−1.24
**D10**	−1.37	−1.16	−1.27
**D11**	−1.34	−1.36	−1.35
**D12**	−1.33	−1.18	−1.26

**Table 2:** Thermal analysis of dendrimers **1–12**.

Dendrimer	Weight Loss (%)	T_onset_ °C	T_endset_ °C
first–generation D1	20	190	220
	60	345	350
	10	500	550
first–generation D2	15	200	220
	75	380	580
	10	600	1000
first–generation D3	15	200	220
	70	390	550
	15	560	620
second–generation D4	30	195	225
	35	355	530
	25	680	750
second–generation D5	25	190	250
	35	400	500
	10	800	990
second–generation D6	20	190	230
	20	420	500
	30	850	900
third–generation D7	30	205	230
	25	406	510
	25	800	980
third–generation D8	20	200	210
	70	300	500
	10	600	1000
third–generation D9	25	200	220
	45	420	500
	15	880	990
fourth–generation D10	15	200	235
	50	340	495
	15	630	790
fourth–generation D11	20	210	230
	70	250	1000
	10	1000	1000
fourth–generation D12	30	220	240
	25	380	480
	10	700	900

**Table 3:** Microbial activity of dendrimers **D1**–**D12**.

Dendrimer	MRSA		VRE		*Staphylococcus warneri*	
	IC_50_ (μM)	MIC (μM)	IC_50_ (μM)	MIC (μM)	IC_50_ (μM)	MIC (μM)
Cp-Fe^II^-arene^71^	28.9	77.5	–	–	38.8	77.5
first-generation D1	3.47 ± 0.00	16	5.07 ± 0.65	32	3.54 ± 0.07	16
first-generation D2	2.32 ± 0.31	16	4.19 ± 0.53	64–32	2.51 ± 0.80	16–8
first-generation D3	3.04 ± 0.00	16	6.08 ± 0.37	32	2.99 ± 0.80	16
second-generation D4	2.99 ± 0.04	8	3.03 ± 0.09	16	2.21 ± 0.23	16–8
second-generation D5	1.11 ± 0.09	32–16	2.15 ± 0.21	64	1.14 ± 0.11	32–16
second-generation D6	2.70 ± 0.41	8	2.64 ± 0.00	32	1.74 ± 0.23	16–8
third-generation D7	0.97 ± 0.04	16	2.95 ± 0.00	32	2.07 ± 0.31	32
third-generation D8	0.65 ± 0.19	32	1.69 ± 0.22	64	0.86 ± 0.02	32
third-generation D9	0.79 ± 0.40	64–32	2.48 ± 0.06	64	1.24 ± 0.07	32
fourth-generation D10	0.82 ± 0.02	16	1.93 ± 0.01	32	1.22 ± 0.02	16
fourth-generation D11	0.53 ± 0.30	64–32	1.02 ± 0.02	64	0.73 ± 0.21	64
fourth-generation D12	0.57 ± 0.03	16	1.62 ± 0.02	64	0.97 ± 0.03	16
paracetamol	>128		>128		>128	
vancomycin	0.6 ± 0.10	1.4			0.5 ± 0.10	0.7
rifampicin			3.5 ± 0.85	4.9		

The dendrimers were tested at eight different concentrations obtained by serial dilution of the initial concentration, 128 µg/mL, to a final concentration 1 µg/mL, in 2% DMSO.
